# Top canopy nitrogen allocation linked to increased grassland carbon uptake in stands of varying species richness

**DOI:** 10.1038/s41598-017-08819-9

**Published:** 2017-08-16

**Authors:** Alexandru Milcu, Arthur Gessler, Christiane Roscher, Laura Rose, Zachary Kayler, Dörte Bachmann, Karin Pirhofer-Walzl, Saša Zavadlav, Lucia Galiano, Tina Buchmann, Michael Scherer-Lorenzen, Jacques Roy

**Affiliations:** 1 CNRS, Ecotron (UPS-3248), Campus Baillarguet, F-34980 Montferrier-sur-Lez, France; 2 Centre d’Ecologie Fonctionnelle et Evolutive, CEFE-CNRS, UMR 5175, Université de Montpellier – Université Paul Valéry – EPHE, 1919 route de Mende, F-34293 Montpellier Cedex 5, France; 3 Institute for Landscape Biogeochemistry, Leibniz-Centre for Agricultural Landscape Research (ZALF), 15374 Müncheberg, Germany; 4 Swiss Federal Research Institute WSL, Zuercherstrasse 111, Birmensdorf, 8903 Switzerland; 5 Berlin-Brandenburg Institute of Advanced Biodiversity Research (BBIB), Altensteinstrasse 6, Berlin, 14195 Germany; 6 UFZ, Helmholtz Centre for Environmental Research, Department Physiological Diversity, Permoserstrasse 15, 04103 Leipzig, Germany; 7 German Centre for Integrative Biodiversity Research (iDiv) Halle-Jena-Leipzig, Deutscher Platz 5e, 04103 Leipzig, Germany; 8 University of Freiburg, Faculty of Biology, Geobotany, Schaenzlestr. 1, D-79104 Freiburg, Germany; 9 Institute of Agricultural Sciences, ETH Zurich, Universitaetsstrasse 2, 8092 Zurich, Switzerland; 10 Slovenian Forestry Institute, Department of Forest Physiology and Genetics and Department of Forest Yield and Silviculture, Večna pot 2, SI-1000 Ljubljana, Slovenia; 11 UFZ, Helmholtz Centre for Environmental Research, Department of Community Ecology, Theodor-Lieser-Strasse 4, 06120 Halle, Germany; 12 Department of Soil and Water Systems, University of Idaho, 875 Perimeter Dr., Moscow, 83844 ID USA

## Abstract

Models predict that vertical gradients of foliar nitrogen (N) allocation, increasing from bottom to top of plant canopies, emerge as a plastic response to optimise N utilisation for carbon assimilation. While this mechanism has been well documented in monocultures, its relevance for mixed stands of varying species richness remains poorly understood. We used 21 naturally assembled grassland communities to analyse the gradients of N in the canopy using N allocation coefficients (*K*_*N*_) estimated from the distribution of N per foliar surface area (K_N-F_) and ground surface area (K_N-G_). We tested whether: 1) increasing plant species richness leads to more pronounced N gradients as indicated by higher K_*N*_-values, 2) *K*_*N*_ is a good predictor of instantaneous net ecosystem CO_2_ exchange and 3) functional diversity of leaf N concentration as estimated by Rao’s Q quadratic diversity metric is a good proxy of *K*_*N*_. Our results show a negative (for K_N-G_) or no relationship (for K_N-F_) between species richness and canopy N distribution, but emphasize a link (positive relationship) between more foliar N per ground surface area in the upper layers of the canopy (i.e. under higher K_N-G_) and ecosystem CO_2_ uptake. Rao’s Q was not a good proxy for either *K*_*N*_.

## Introduction

During the last two decades, substantial progress has been made in understanding the role of biodiversity for ecosystem functioning^1–3^. Numerous meta-analyses^4, 5^ and biodiversity experiments such as the Cedar Creek experiment^6^, the pan-European Biodepth experiment^7^ and the Jena Experiment^8, 9^ have attested to a positive relationship between plant diversity and ecosystem functioning. However, while there is now consensus that biodiversity begets productivity due to complementarity^10, 11^, insurance^12, 13^ and selection effects^14^, the underlying physiological mechanisms are not fully understood. Furthermore, there is increasing evidence that functional diversity is of greater importance for ecosystem functioning than the number of taxonomic species^15, 16^. Several functional diversity metrics currently quantify the variety, range and evenness of community traits and assume that a greater dissimilarity of traits indicates less niche overlap, and hence, more efficient capture of resources in time and space^17–19^. Two recent studies^20, 21^ found the diversity of leaf nitrogen (N) concentration in the canopy, as measured by Rao’s quadratic entropy (FD_Q_-N)^18^, to be a reliable predictor of net ecosystem CO_2_ exchange (NEE), gross primary productivity and water use efficiency in grassland communities. The authors suggested that FD_Q_-N might be related to the vertical distribution of N in the canopy, with higher FD_Q_-N values indicating canopies with more pronounced N gradients in the canopy increasing from bottom to the top of the canopy. This conjecture is in line with the optimal N allocation hypothesis^22–24^, which states that a link between canopy N distribution and CO_2_ uptake should be expected because canopies with increasing leaf N concentrations from bottom to top of the canopy, following the corresponding increase in light availability, should be more optimal. Such more optimal canopies should have higher nitrogen use efficiencies (NUE) and photosynthetic carbon (C) uptake because the gain in C uptake per unit N investment is greater at higher light intensities for a given N content^23^. However, to date, the evidence for a link between plant diversity, canopy N allocation and canopy-level CO_2_ uptake remains circumstantial and has not been directly tested. Furthermore, the majority of studies that investigated the links between canopy N distributions and ecosystem C uptake used computer simulations, simplified communities (i.e., monocultures)^22, 24, 25^ or used controlled environmental conditions with small containers, hydroponics or artificial light^26–28^, which cautions against the generality of their results.

Canopies of monocultures have often been found to have more uniform than optimal N distributions, but a closer to optimal N allocation has been found to lead to 1–42% higher C uptake due to increased NUE^22–24, 29^. In mixed stands of varying species richness, where dominant and subordinate species emerge, it is less clear how the interspecific competition for light affects the N allocation profiles and, consequently, its impact on the ecosystem C uptake. Wacker *et al*. (2009)^30^ suggested that mixed stands may be more efficient in C uptake than monoculture stands because they form “integrated” canopies with complementary allocation of leaf mass and nitrogen along the vertical light profile, and that canopies including species with a larger range of leaf mass and N values may have a higher likelihood of assembling “integrated” canopies. However, several theoretical studies suggested the contrary, namely that mixed stands may be less efficient in C uptake due to a competition driven “tragedy of the commons” owing to the fact that the most competitive strategy at the individual level is to maximise its own C gain. This can result in suboptimal light utilisation across the entire canopy in multispecies stands^31, 32^. Yet, multispecies stands have often been found to be more productive in biodiversity experiments^33, 34^. Therefore, it is currently unclear whether species-rich stands are more efficient at matching the foliar N content with the emerging light attenuation profiles, and thus, achieve more pronounced vertical gradients of foliar N allocation leading to increased C gain.

To fill the aforementioned knowledge gaps, we took advantage of 21 communities varying in plant species richness that assembled naturally after cessation of weeding in subplots of the Jena Experiment^35^, *i*.*e*. where the control of species richness and composition was abandoned for at least seven years. Specifically, we test the following hypotheses: 1) increasing plant species richness leads to more pronounced vertical N gradients estimated with the N allocation coefficients (*K*_*N*_) according to the method of Hirose and Werger (1987)^23^, 2) K_N_ is a good predictor of instantaneous NEE and 3) functional diversity of leaf N concentration as estimated by Rao’s Q quadratic diversity metric is a good proxy of K_N_ (see Table 1 for a list of abbreviations). To this end we tested two versions of K_N_, one based on the distribution of foliar N content per leaf surface area (*K*_*N-F*_), the other based on the distribution of foliar N content per ground surface area (*K*_*N-G*_).

**Table 1:** Table explaining the most important abbreviations.

Abbre-viation	Description	Unit
F_BM_	Foliar biomass (DW) per ground surface area	g DW m^−2^
FD_Q_-N	Functional diversity of leaf N concentrations calculated based on species-level averaged foliar N	unitless
K_L_	Canopy light attenuation coefficient (eq. 2)	unitless
K_N-F_	Nitrogen allocation coefficient in the canopy (eq. 1) calculated based on canopy N amount per foliar surface area	unitless
K_N-G_	Nitrogen allocation coefficient in the canopy (eq. 1) calculated based on the N amount per ground surface area	unitless
LAI	Total leaf area index	m^2^ leaf m^−2^ ground
LAI_D_	Cumulative LAI from the top of the canopy to the depth D	m^2^ leaf m^−2^ ground
LUE	Light use efficiency	µmol CO_2_ m^−2^ s^−1^/ µmol PAR m^−2^ s^−1^
Mixed	Mixed stands containing several (2 to 5) dominant plant species	type of stand
Mono	Stands dominated by a single plant species	type of stand
NEE	Instantaneous net ecosystem CO_2_ exchange at canopy level	µmol CO_2_ m^−2^ s^−1^
N_F_	Foliar N per leaf surface area	g N m^−2^ leaf
N_G_	Foliar N per ground surface area	g N m^−2^
N_R_	N content present in reproductive organs (mainly inflorescences)	g N m^−2^
NUE	Nitrogen use efficiency	µmol CO_2_ m^−2^ s^−1^/g N m^−2^
PAR	Photosynthetic active radiation measured at the top of the canopy	µmol m^−2^ s^−1^
RSR	Realised species richness including all species present (See Table S1).	count
SR15	Species richness including species with a ground surface cover higher than 15% (See Table S1).	count
T	Air temperature in the cuvette used for NEE measurements	°C

## Results

Since the NEE results indicated a statistically significant difference between monospecific-dominated and mixed stands, but no plant diversity effects, we focus our presentation of the results on emphasizing differences between monospecific-dominated and mixed stands.

### Vertical distribution of foliar nitrogen and light in the canopies

**Figure 1:**
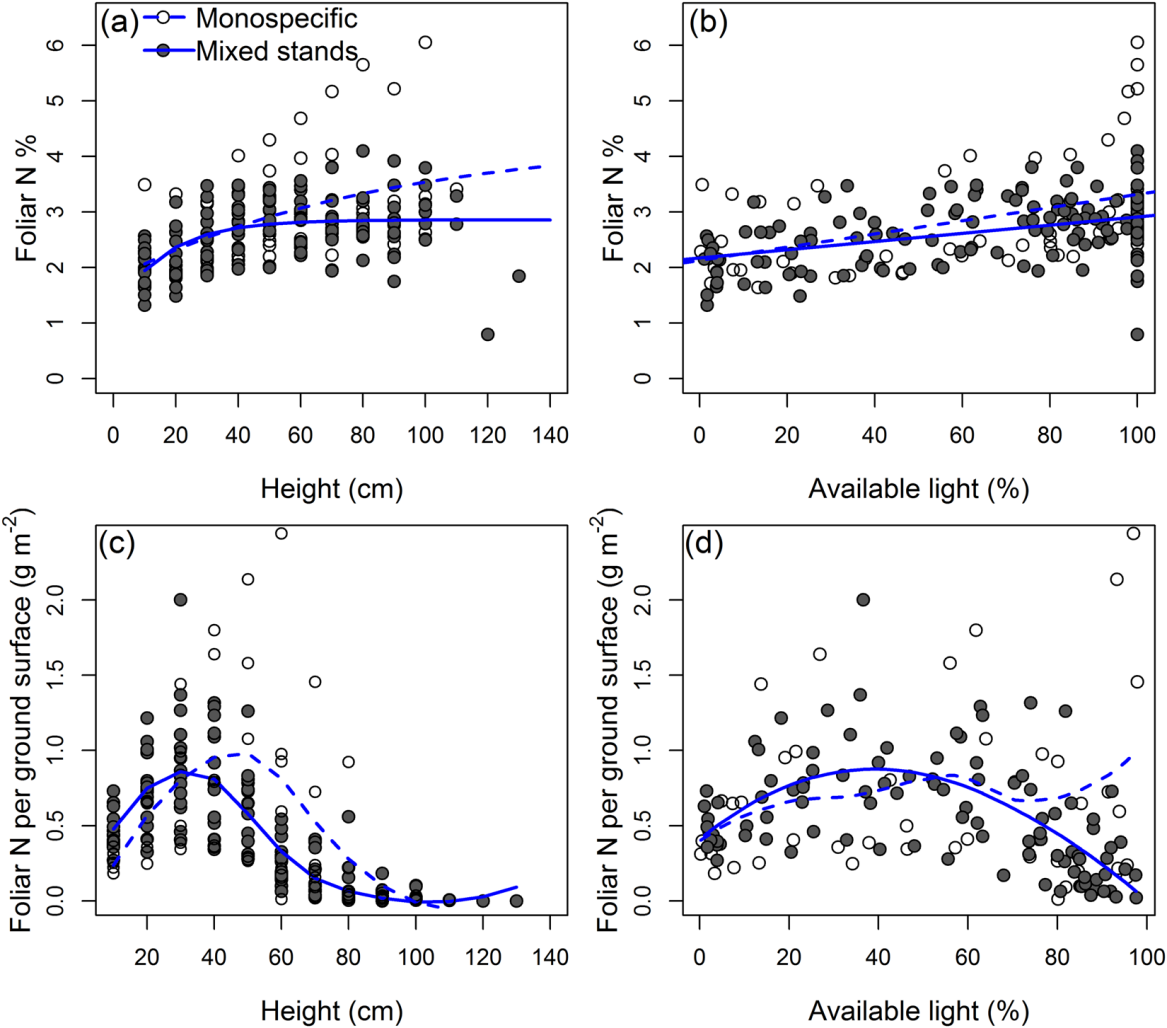
Relationship between the foliar nitrogen (N) concentration and (**a**) height in the canopy and (**b**) percentage of light transmission in monospecific-dominated and mixed stands. Relationship between leaf N content per ground surface area and (**c**) height in the canopy and (**d**) percentage of available light.

The relationship between the canopy height and the percentage of foliar N was best described by a 3-parameter exponential asymptotic, showing an increase of foliar N concentrations with height, which levels off at the top of the canopy. Based on the intercepts resulting from our exponential function fit to the data, foliar N concentrations in the lowest layer were predicted to be higher in the monospecific-dominated stands relative to mixed stands (1.78 *vs*. 1.16% N intercept; Fig. 1a). The coefficients indicating the saturating N concentrations towards the top of the canopies were 4.31% and 2.85% N for monospecific-dominated and mixed stands, respectively (Fig. 1a). The percentage of foliar N also increased linearly with the percentage of available light (Fig. 1b), with a slightly higher slope for monospecific-dominated stands (N% = 2.13 + 0.012*%light, R^2^ = 0.19, P < 0.0001) relative to mixed stands (N% = 2.17 + 0.007*%light, R^2^ = 0.20, P < 0.0001). The light attenuation coefficients (*K*_*L*_) varied from 0.56 to 0.90, with a mean of 0.67, and a median of 0.64 (see Supplementary Fig. S1). *K*_*L*_ values were not significantly different between monospecific and mixed stands (F_1, 19_ = 0.02, P = 0.875) and no significant effect of RSR (F_1,19_ = 0.83, P = 0.371) or SR15 (F_1,19_ = 0.13, P = 0.724) on *K*_*L*_ was found. Similarly, no significant effect of the type of stand on LAI was found (5.99 vs. 5.57 for monospecific and mixed stands respectively; F_1.19_ = 0.30, P = 0.587). Furthermore, no significant effect of RSR (F_1.19_ = 0.44, P = 0.512) or SR15 (F_1.19_ < 0.01, P = 0.979) were observed.

The relationship between the foliar N content per ground surface area (g N m^−2^ ground surface; N_G_) and the height in the canopy as well as the available light exhibited a humped shape (Fig. 1c). The distribution of N_G_ in the canopy differed between the monospecific-dominated and mixed stands. Monospecific-dominated stands exhibited lower N_G_ at 10 (−39%, t = −4.05, P = 0.001) and 20 cm (−39%, t = −2.24, P = 0.037) height and three- to four-fold higher N_G_ in the higher strata at 60 (t = 2.74, P = 0.013) and 70 cm (t = 2.22, P = 0.039) height.

### Nitrogen allocation coefficients (*K*_*N*_)

**Figure 2:**
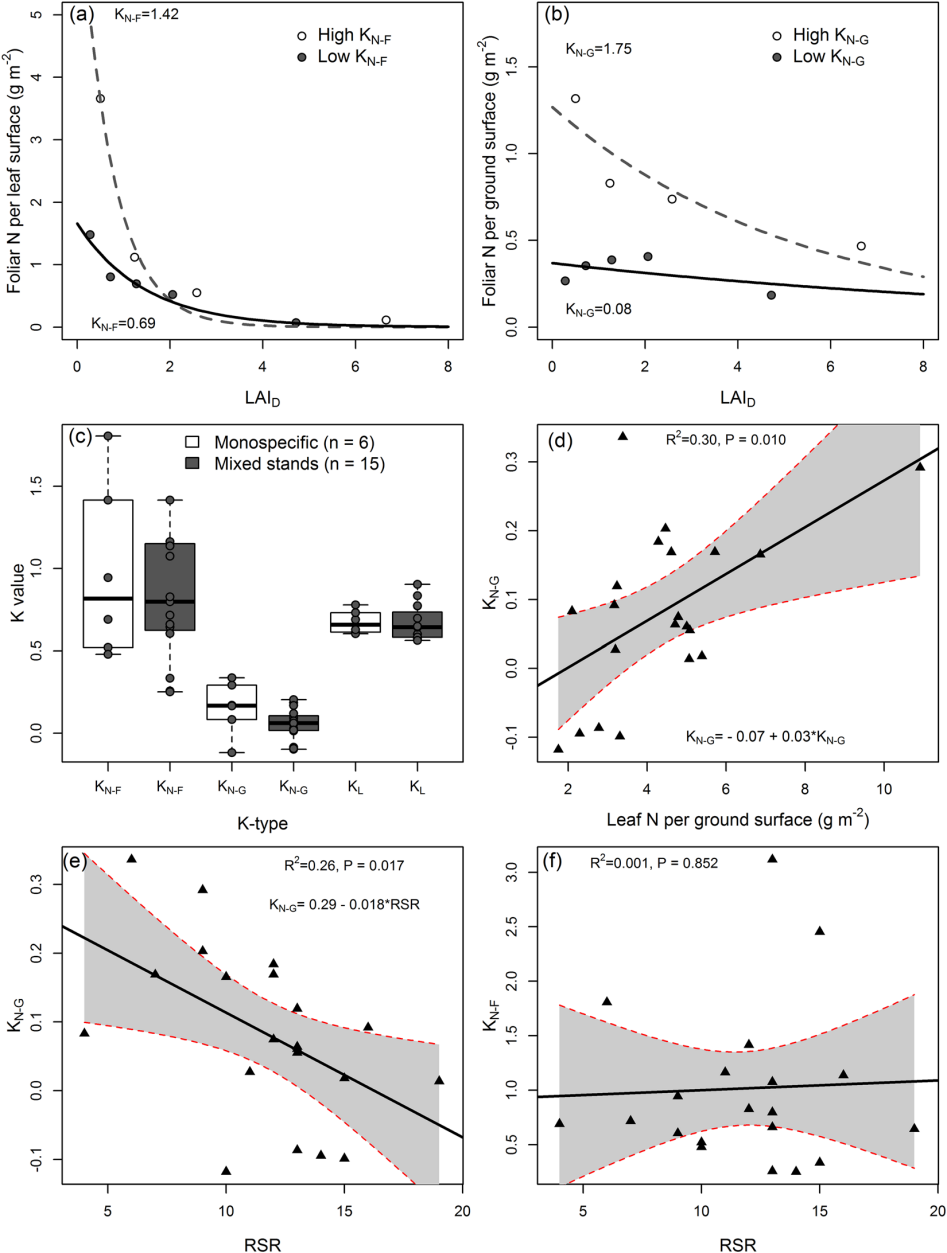
(**a**) Examples of two plots (no. 1 and 21 from Table S1) with contrasting K_N-F_ values estimated with eq. (2) from the relationship between foliar N per leaf surface area and leaf area index at different depths (LAI_D_) (**b**) Same as for Fig. 2a but for the N allocation coefficient based on foliar N per ground surface area (*K*_*N-G*_). (**c**) Boxplots depicting the median and distributions of *K*_*N-F*_, *K*_*N-G*_ and light attenuation coefficients (*K*_*L*_) in monospecific and mixed stands. (**d**) Relationship between the total foliar N content per ground surface area (N_G_) and *K*_*N-G*_. (**e**) Relationship between realised species richness (RSR) and *K*_*N-G*_. (**f**) Relationship realised species richness (RSR) and *K*_*N-F*_.

To help visualising how the K_N-F_ and K_N-G_ values were obtained, in Fig. 2a and b we show examples of two plots with contrasting *K*_*N-F*_ and *K*_*N-G*_ values (plots no. 1 and 21 from Table S1) derived from the relationship between foliar N content (per ground or foliar surface) and the LAI at different canopy heights using eq. (2) (see Supplementary Figs S2 and S3 to see the fitted values for *K*_*N-F*_ and *K*_*N-G*_ in all plots). Across the 21 communities, the values of *K*_*N-F*_ ranged between 0.25 and 3.12, with an overall mean of 1.01 and a median of 0.79. No significant relationship between *K*_*N-F*_ and the tested predictors (species richness, type of stand, LAI, N_G_, and K_L_) was found.

In contrast to *K*_*N-F*_, *K*_*N-G*_ showed lower values, ranging from −0.11 to 0.33, with an overall mean of 0.08 and a median of 0.07 (see Supplementary Fig. S3). *K*_*N-G*_ was best predicted by models incorporating leaf N content per ground surface area (N_G_, with a positive coefficient, Fig. 2d) and realised species richness (RSR, with a negative coefficient, Fig. 2e) (Table 2). No effect of Simpson, Shannon diversity metrics nor the evenness metric was found on any of the two N allocation coefficients (Supplementary Fig. S6).

**Table 2:** The five candidate models explaining the nitrogen (N) distribution coefficient per ground surface area (K_N-G_) as a function of light attenuation profiles (K_L_), realised species richness (RSR), total leaf N (N_G_) and the N content allocated to reproductive organs (N_R_).

K_N-G_		AICw	AICc	R^2^	P-value
1.	K_N-G_ = 0.16 + 0.05*K_L_*N_G_ − 0.03*K_L_*RSR	–36.61	0.13	0.56	>0.001
2.	K_N-G_ = 0.14 + 0.03*N_R_*N_G_ − 0.02*N_R_*RSR	–36.30	0.11	0.56	>0.001
3.	K_N-G_ = 0.14 + 0.03*N_G_ − 0.02*RSR	–36.15	0.10	0.56	>0.001
4.	K_N-G_ = 0.13 + 0.03*N_G_ − 0.02*RSR*K_L_	–36.11	0.10	0.55	>0.001
5.	K_N-G_ = 0.27–0.03*RSR + 0.003*RSR*N_G_	–35.90	0.09	0.55	>0.001

No significant predictors were found for K_N-F_. AICc represents the Akaike Information Criterion adjusted for sample size whereas Akaike weights (AICw) represents the probability that a particular model is the best fit to the data. See Table 1 for an abbreviation list.

### Relationship between stand carbon uptake and leaf nitrogen allocation

**Figure 3:**
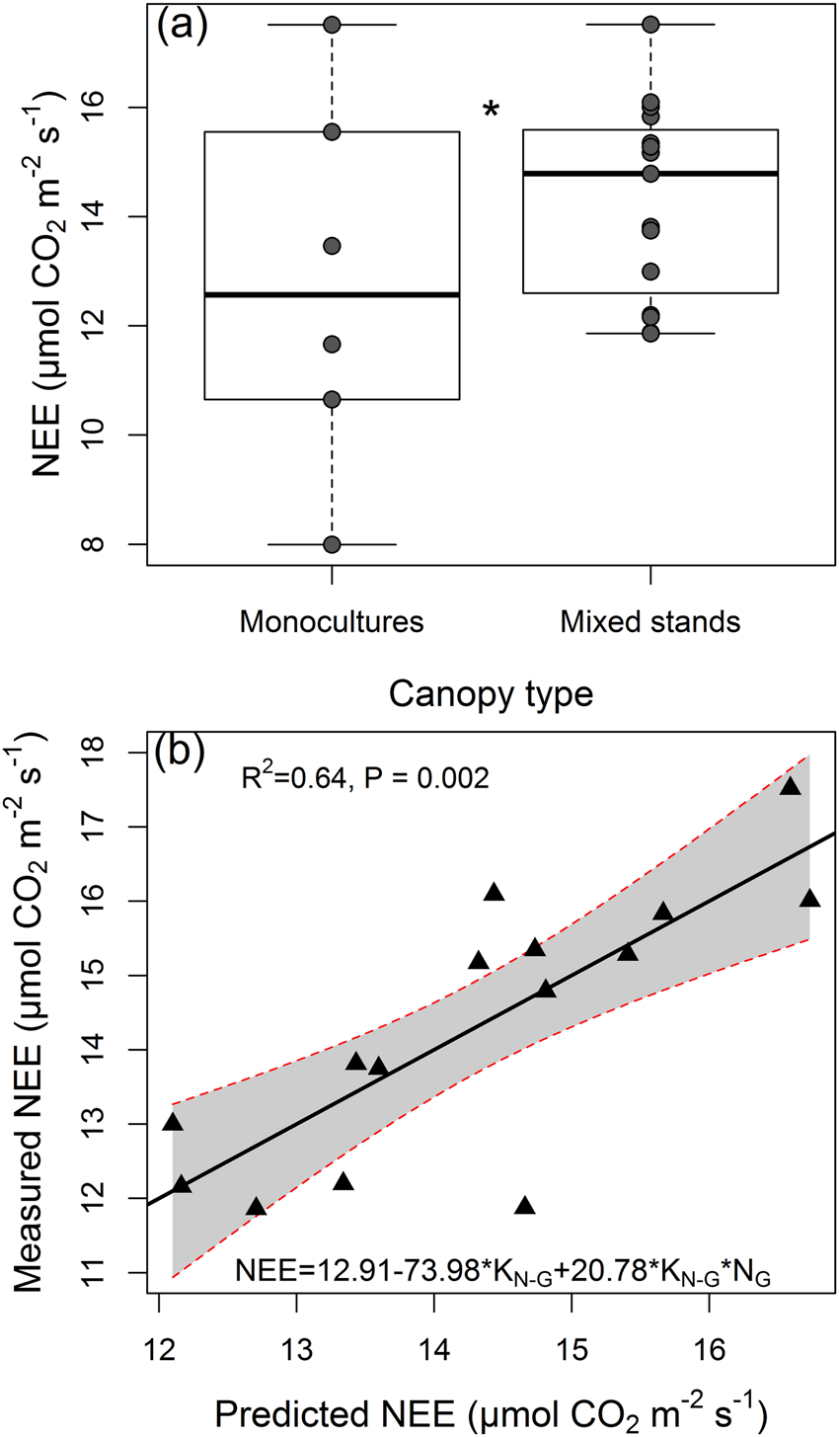
(**a**) Boxplots showing the median and variation in instantaneous CO_2_ net ecosystem exchange (NEE) measured in monocultures and mixed stands. Multiple regression results from Table 3 show significantly different (P < 0.05, *) regression coefficients for monospecific and mixed stands. (**b**) Relationship between predicted NEE values by the best model from Table 3 and measured NEE in mixed stands.

Multiple regression analyses (Table 3) indicate that NEE was higher in mixed stands relative to monospecific ones (Fig. 3a). No significant effect of RSR or SR15 on NEE was found (Supplementary Fig. S5). Similarly, no significant effect of Shannon and Simpson diversity metrics or community evenness affected NEE (Supplementary Fig. S6). As we found a significant increase of NEE in mixed stands concomitant with a tendency of lower K_N-G_ values, we further analysed the relationship between the N allocation coefficients and NEE in all communities as well as in mixed stands only.

**Table 3:** The five best models predicting the instantaneous CO_2_ net ecosystem exchange (NEE) as a function of nitrogen (N) allocation confidents (K_N-F_ and K_N-G_), foliar biomass (F_BM_), percentage of legume cover (Leg) and type of stand (Mono = monospecific-dominated stands, Mix = mixed stands).

No	Model	AICc	AICw	R^2^	P-value
All stands (n = 21)					
1	NEE = 13.81 + 2.88*Leg*Mono + 1.99*Leg*Mix − 0.16*Leg*F_BM_	63.92	0.41	0.59	0.001
2	NEE = 14.17 + 2.57*Leg − 0.02*Leg*F_BM_	65.75	0.17	0.48	0.003
3	NEE = 6.87 + 0.059*F_BM_ − 0.003*N_G_*F_BM_	66.34	0.02	0.37	0.015
4	NEE = 8.56 + 0.03* F_BM_ − 0.17*Leg*N_G_	66.70	0.02	0.37	0.015
5	NEE = 8.05 + 0.04* F_BM_ − 1.78* F_BM_*K_N-G_	67.79	0.02	0.37	0.016
Mixed stands only (n = 15)					
1	NEE = 12.91–73.98*K_N-G + _20.78*K_N-G_*N_G_	61.32	0.18	0.64	0.002
2	NEE = 13.43–48.89*K_N-G_ + 0.34*K_N-G_*F_BM_	61.61	0.16	0.47	0.020
3	NEE = 13.19–0.005 *Leg*F_BM_	63.61	0.06	0.21	0.084
4	NEE = 9.71 + 0.02* F_BM_ − 0.006 *Leg*F_BM_	63.67	0.06	0.38	0.053
5	NEE = 8.23 + 0.03* F_BM_ − 0.94*Leg	63.80	0.05	0.37	0.059

AICc represents the Akaike Information Criterion adjusted for sample size whereas Akaike weights (AICw) represents the probability that a particular model is the best fit to the data. See Table 1 for a list of all abbreviations.

When the monoculture and mixed stands were analysed together, the model explaining best the NEE (R^2^ = 0.59, P = 0.001) included an interaction between the percentage of legumes in the canopy and type of stand as well as an interaction between the percentage of legumes and canopy leaf biomass (F_BM_) (Table 3). Overall, F_BM_ was a consistent predictor of NEE. *K*_*N-G*_ was also retained among the five best models. However, when we focused on mixed stands only, the best model predicting NEE incorporated *K*_*N-G*_ and an interaction between *K*_*N-G*_ and N_G_ (R^2^ = 0.64, P = 0.002; Table 3 and Fig. 3b).

### Effects of N allocation on the light and nitrogen use efficiencies of NEE

To explore the potential relationships between canopy N allocation and the nitrogen use efficiency (NUE) and light use efficiency (LUE) of instantaneous NEE while avoiding the intrinsic species-specific differences occurring in monocultures, we focused on mixed stand only. LUE was best predicted by a model that included a positive coefficient for *K*_*N-G*_ as well as a negative temperature (T) effect (LUE = 0.018 − 0.0003*T + 0.0015*K_N-G_*N_G_; P = 0.001, R^2^ = 0.68). In contrast, the model for nitrogen use efficiency (NUE) was best predicted by *K*_*N-G*_, with a negative regression coefficient and the interaction between temperature and the percentage of legumes in the canopy, with a negative coefficient (NUE = 8.80 − 0.88*N_*G*_ − 0.008*LAI*T; P < 0.001, R^2^ = 0.84). See Supplementary Fig. S7 for a scatterplot and correlation matrix depicting the relationships between NUE, LUE and their most important predictors.

## Discussion

To our knowledge, this is the first study assessing the potential links between canopy N allocation coefficients (K_N_) and net ecosystem CO_2_ exchange (NEE) in stands of varying species richness under field conditions. Concerning our first hypothesis, we found that increasing plant species richness did not lead to canopies with higher N distribution coefficients compared to monospecific-dominated stands. Instead, we found that increasing the number of species present in the canopy leads to lower K_N-G_ values, meaning that relatively less N was allocated at the top of the canopy where light availability is higher at higher diversity levels. These results are in line with the studies of Anten^29^ and Wacker *et al*.^30^ documenting that species in mixed stands exhibit non-optimal N allocation for increasing C gain at the whole stand level. The latter study suggested that optimal N profiles in mixed stands emerge only if the coexisting species had already complementary N profiles based on the among-species variation in monocultures. This can be explained by the fact that N cannot be allocated between species (e.g. a small size species with high N content cannot offer N to a tall species), while N can be allocated to different leaves and heights within an individual. Alternatively, Hikosaka (2014) proposed that lower *K*_*N*_ values could result from a lower predictability of light availability in dense canopies. This could be a potential explanation for the decreasing K_N-G_ values with increasing species richness as the competition for light over time and during stand development might be less predictable in species rich communities compared to monospecific stands. Therefore, individual plants in species rich stands might be less able to project which leaves will receive more direct light than others. In other words, if the position and duration of direct light is less predictable in species rich stands, plants will benefit less from allocating more N towards the upper layers of the canopies^36^. Taken together, our results support the tragedy of the commons phenomena^29, 31^, in which light acquisition strategies of species optimise for the individual rather than for the entire canopy.

Although vertical gradients of leaf N allocation are a common feature of plant canopies, the factors controlling the formation of non-uniform N distributions are still debated. Theory predicts that canopy N profiles are the result of the interaction between N availability and the light attenuation profiles^22, 23, 37^. Several studies suggested that more pronounced gradients of N allocation in the canopy (i.e. higher *K*_*N*_ values) will be achieved if more N is available for allocation and redistribution, and a positive correlation between total canopy N content and *K*_*N*_ has been reported^23, 38^. However, two other studies^26, 28^ showed an inverse trend, namely a more uniform N allocation with increased N supply, but these two studies where performed in small pots/containers grown in greenhouses and growth chambers with artificial lighting, conditions arguably different from natural field conditions. In our study, with canopies naturally assembled in the field, we found that *K*_*N-G*_ was consistently predicted (with positive regression coefficients, Table 2) by the total amount of foliar N per ground surface area (N_G_). However, the attenuation coefficients (*K*_*L*_) also proved to be important for *K*_*N-G*_ and, as predicted by theoretical work^22, 24, 37^, the interaction between *K*_*L*_ and N_G_ was also a significant predictor of *K*_*N-G*_ (Table 2). Overall, our results support the theoretical findings of Hirose and Werger (1987)^23^ indicating that the allocation of N in the canopy is influenced by the interaction between N availability and light extinction profiles.

Two recent papers put forth the hypothesis that a more optimal N allocation in the canopy could occur in mixtures with higher species richness, and that this might be part of the mechanistic basis to explain the higher C and water fluxes observed in higher diversity mixtures^20, 21^. The supporting evidence for this conjecture relied on the detected positive correlations between carbon and water fluxes and a functional diversity metric (Rao’s quadratic entropy of leaf N concentrations, FD_Q_-N), which captures the diversity/dissimilarity of foliar N concentrations between the species of a mixture. Here, we directly tested the hypothesis that that FD_Q_-N could be a good proxy of *K*_*N*_ and found no supporting evidence that FD_Q_-N is positively correlated with *K*_*N-F*_ and K_N-G_. However, one caveat needs to be noted relative to the previous studies proposing FD_Q_-N as a good proxy of *K*_*N*_. In this study, the calculation of FD_Q_-N was based on values of foliar N measured in plants collected *in situ* but not directly from the plots included in our experiment. Although the literature is abundant with functional trait-based metrics calculated with literature-derived or non *in-situ* measured trait values^39–41^, the fact that FD_Q_-N was calculated with foliar N values not measured in our communities remains a notable caveat in this study because there is evidence that plant species change their trait values in response to the growth conditions experienced in mixtures varying in diversity^42, 43^.

Up to now, the impact of N allocation on canopy-level C gain relied exclusively on canopy models to upscale the relationship between leaf photosynthetic capacity (Amax) and leaf N content (Supplementary Fig. S8) to canopy values^23, 37^. Here, we opted to test the relationship between canopy C uptake and N allocation coefficients using instantaneous NEE because midday values of instantaneous NEE measured in the same experimental system were vastly dominated by photosynthetic activity^20^. Although no significant impact of species richness was found on the NEE, our results provide supporting evidence for our third hypothesis postulating that the allocation of N in the canopy is important for whole canopy C gain. Whilst the control of NEE is complex because is affected by multiple factors, including (but not limited to) phenological status, temperature, soil moisture and light intensity, here we found that when more pronounced gradients of canopy N allocation (increasing from bottom to top of the canopy) were found in mixed stands as indicated by higher K_N-G_ values, it led to higher NEE and LUE. This relationship was weaker when monocultures were included, but this can be explained by the intrinsic species-specific photosynthetic efficiencies of monocultures which presumably override the effect of the N allocation coefficients.

One important finding of this study is that of the two estimated N distribution coefficients, the one expressing the canopy N distribution per ground surface area (K_N-G_) was actually more relevant for canopy level C uptake than the distribution of canopy N based on foliar surface area (*K*_*N-F*_) (Table 3). Although there is substantial theoretical and empirical evidence that K_N-F_ (nitrogen allocation coefficient for leaf area-based leaf N) is a reliable predictor of the photosynthetic uptake of individual plant^23, 24, 44^ and monospecific stands, its predictive power for stand-level C fluxes in mixed stands was not directly demonstrated by *in-situ* measurements. However, *K*_*N-F*_ does not take into account the leaf biomass distribution by height, and consequently, a high N content per leaf area at the top of the canopy leading to a high K_N-F_ value only indicates that the leaves at the top of the canopy have high N content, but it does not indicate the amount of foliar biomass, an important determinant of stand-level C uptake. In contrast, K_N-G_ takes into account the leaf biomass distribution by height as a high N content per ground surface area at the top of the canopy leading to a high K_N-G_ value indicates that there is a significant amount of foliar biomass and foliar N at the top of the canopy where light is less limiting. Furthermore, we argue that K_N-F_ is prone to a strong bias introduced by the N concentration of the leaves situated at the very top of the canopy which often represent a minor proportion of the total LAI, and therefore, will have a relatively low contribution to the overall canopy level photosynthetic activity. The K_N-F_ values can be overinflated when the leaves of the top of the canopy have disproportionally higher N concentrations relative the rest of the canopy. An underestimation of the N gradient in the canopy can also occur when the top leaves have disproportionally lower N due to reallocation to reproductive organs. K_N-G_, on the other hand, captures the distribution of total foliar N per ground surface area across the height of the whole canopy, and our results indicate that is a much better predictor of stand-level NEE than K_N-F_.

In conclusion, using naturally assembled canopies varying in species richness in field conditions, this study shows that increasing species richness leads to less pronounced vertical gradients (increasing from top to bottom of the canopy) of N allocation in the canopy. However, when pronounced vertical gradients of N allocation (i.e. with higher K_N-G_ values) occurred in the canopies of mixed stands, the positive relationship between K_N-G_ values and canopy carbon uptake hold true, leading to increased canopy-level NEE and LUE. This knowledge can be important in the selection of high-performance intercropping systems with more optimal N allocations in the canopy, which will help to increase productivity.

## Methods

### Field site and sampling strategy

The site of the Jena Experiment (50° 57.1′ N, 11° 37.5′ E, 130 m above sea level; mean air annual temperature 9.3 °C, mean annual precipitation 587 mm^45^, is located on the floodplain of the Saale River (Jena, Germany), and was a former arable field until 2000. After two years of fallow, in May 2002, 82 plots (20 × 20 m) varying in sown plant species richness (1, 2, 4, 8, 16 and 60 species), plant functional groups (1 to 4, grasses, small herbs, tall herbs and legumes), and plant species composition were established^46^. The 82 plots were randomly allocated to four blocks that were identified to vary in soil texture^46^. Sown plant species composition was sustained by regular weeding of all unwanted species. This study was performed in 21 subplots (4.5 × 6.5 m) with plant communities designated initially for an invasion/colonisation experiment^47^ and which, at the time of our study, were not weeded for seven years. These subplots were preferred to the maintained experimental gradients of species richness of the Jena Experiment because we sought communities with self-assembled intact canopies that were not disturbed by weeding. Furthermore, these communities assembled under similar environmental conditions after the initial sowing, had all developed closed canopies, but were still different in species richness, composition and functional diversity^35^, which was an important prerequisite to test our hypotheses. A representative area of 1 m^2^ was selected in each plot and used for the measurements of leaf area index (LAI) and light extinction profiles, while a central circular area of 0.196 m^2^ in each plot was used for NEE, aboveground biomass measurements and species abundances (estimated based on surface cover using a modified decimal scale; <1%, 1–5%, 6–15%, 16–25%, etc.). As the community composition in the invasion plots was not perfectly homogenous, the selected representative areas (of 1 m^2^) were selected following two a priori defined criteria: 1) to avoid areas that had a strongly different community composition than the majority of the selected plot area, and 2) communities developed closed canopies, i.e. with minimum surface of bare ground. These communities contained between 4 and 19 realised species richness (RSR) near equally distributed in 3 of the 4 blocks of the Jena Experiment (8 plots from Block I, 6 plots from Block II and 7 plots from Block III). In addition to RSR, we derived an additional species richness metric by including species with a surface cover higher than 15% (henceforth SR15). We argue that this threshold is relevant to canopy performance measurements such as the instantaneous CO_2_ net ecosystem exchange (NEE) which is controlled by the dominant species of a canopy. SR15 was also used to define monospecific (n = 5) and mixed stands (n = 15); see also Supplementary Table S1. In addition to RSR and SR15 we also present the results for three classical diversity metrics (Simpson, Shannon and Pielou’s evenness) as supplementary materials.

### Measurements of net ecosystem CO_2_ exchange

The relationship between canopy C uptake and N allocation coefficients was analysed using instantaneous (NEE), which represents the difference between gross primary production (GPP) and ecosystem respiration (Reco). Whilst GPP (where GPP = NEE − Reco) represents the actual canopy C uptake, we used NEE as a proxy for canopy C uptake because midday values of NEE measured in the same experimental system were vastly dominated by GPP (due to relatively low values of Reco)^20^. Two simultaneously calibrated (with the same CO_2_ calibration gas) infra-red gas analysers (IRGAs), one LI-6400 (LI-COR Environmental, USA), and one Walz GFS-3000 (Heinz Walz GmbH, Germany) were used in connection with two large cylindrical (0.5 m dia. and 1.3 m height) UV-VIS transparent cuvettes made of polycarbonate to measure NEE. The measurements of CO_2_ drawdown were used to calculate the instantaneous NEE as µmol CO_2_ per m^−2^ s^−1^ using the ideal gas law. For these measurements, the chambers were closed and the IRGAs were operated in a closed loop setup for 5 minutes (*cf*. Volkmann *et al*.^48^. The measurements were performed during two consecutive days (20^th^ and 21^st^ of May 2014) with clear sky (i.e. sunny conditions) between 11am and 15 pm local time. During the measurements of NEE, the temperature and pressure in the cuvettes were recorded with the ancillary temperature and pressure sensors of the IRGAs. Incoming radiation values (PAR) where provided by the weather station of the Jena Experiment. Furthermore, the NEE measurements together with radiation and foliar N measurements allowed us to also estimate the light use efficiency (LUE; NEE per available PAR) and nitrogen use efficiency (NUE; NEE per total foliar N) of the measured NEE.

### Measurements of leaf area index (LAI) and light extinction coefficients (K_L_)

An SS1-SunScan canopy analyser coupled with a BF5 sunshine sensor (Delta-T Devices, Cambridge, UK) was used to estimate the light extinction profiles and the leaf area index (LAI) of the canopies. The SS1 canopy analyser was chosen due to its advantageous dimensions (1 m long by 13mm wide probe with PAR sensors each 15.6mm) which allowed for the estimation of LAI and light extinction profile of the selected 1 m^2^ plots at seven heights (at 3, 10, 20, 30, 40, 50 and 60 cm) by averaging 5 readings (20 cm horizontally apart) for each height. This allowed for the estimation of the light attenuation coefficients through the canopy as estimated using the Beer’s law^49^:

1 $$I={Io}\,exp\,(-{K}_{L}\,{{\rm{LAI}}}_{{\rm{D}}})$$

where *Io* and *I* are the photon flux density on a horizontal plane above the canopy and within the canopy, respectively, at a given leaf area index value cumulated from the top of the canopy (LAI_D_); *K*_*L*_, is the extinction coefficient.

### Aboveground plant biomass and N measurements

Following the NEE measurements, the aboveground biomass included in the cuvette was harvested by layer, from the top towards the bottom of the canopy at 10 cm intervals, and separated into leaves, stems and inflorescences. The different components of the biomass were weighed after drying at 70 °C for two days until constant weight. The leaf biomass by layer as well as the pooled stem and inflorescence biomass were milled into a homogeneous powder and analysed for N content using an elemental analyser (Truspec CNS; LECO Instrumente GmbH, Mönchengladbach, Germany).

### Estimation of the nitrogen allocation coefficients (*K*_*N*_)

The measurements of leaf N content by layer were used to estimate the coefficient of N allocation in the canopy (*K*_*N*_) following the method of Hirose and Werger (1987)^23^. The following exponential function was used to estimate *K*:

2 $${{\rm{N}}}_{{\rm{D}}}={\rm{N}}\,\exp (-{K}_{N}\,{{\rm{LAI}}}_{{\rm{D}}})$$

where N_D_ and N represent leaf nitrogen content per unit area within the canopy at depth D and at the top uppermost layer of the canopy, respectively and LAI_D_ represents the cumulative leaf area from the top of the canopy to the depth D. Larger positive *K*_*N*_ values signify a less uniform N allocation in which upper leaves in the canopy have higher N concentrations than lower leaves to match the increased light availability. *K*_*N*_ = 0 indicates a uniform allocation of leaf N in the canopy, in which every leaf has a N content equal to the mean. *K*_*N*_ < 0 signifies that leaves that are lower in the canopy have higher N content. Following this approach two versions of *K*_*N*_ have been estimated. One, where canopy N was expressed per foliar surface area (*K*_*N-F*_), which should capture the vertical distribution of the N per leaf area, and a second in which N was expressed per ground surface area (*K*_*N-G*_). We expect *K*_*N-G*_ to be a better predictor of canopy NEE because it captures the vertical distribution of the total N amount in the canopy. To minimize biasing the *K*_*N*_ values with data points from the highest canopy layers, which have a minimal LAI and hence a minimal contribution to the carbon fluxes but widely varying N concentrations, we only included data when the cumulative LAI from the top of the canopy was higher than 5% of the total LAI.

### Functional diversity metric based on leaf nitrogen concentrations

We used Rao’s quadratic entropy^18^ to calculate an index of diversity of leaf N concentrations present in the canopy which was previously found to be a good predictor of carbon and water fluxes^20, 21^. FD_Q_-N incorporates information about functional distance as well as functional evenness of a community because it is abundance weighted using species-specific plant cover data. In addition to FD_Q_-N, we also estimated functional richness (FRic) representing the amount of functional space filled by the community and functional evenness (FEve) which describes the evenness of abundance distribution of foliar N concentrations^50^. These metrics where calculated based on the average leaf N concentration per species (estimated from pooling leaves from all canopy heights) as measured *in situ* in 2013 (with the exception of three species measured in 2015). They were computed using the “FD” package^17^ available through the R^51^ statistical package version 3.1.3.

### Statistical analyses

All statistical analyses were performed in R statistical package version 3.1.3. To describe the shape of the relationship between the height of the canopy and the leaf N% (or N content per surface area) on the graphs we tested linear (y = *a* + *bx*), 2-parameter (*y* = *a* (−*e* ^−*bx*^)) and 3-parameter (*y* = *a* − *b*e^−*cx*^) exponential functions as well as locally weighted scatter-plot smoothers (loess). The exponential functions were fitted within the “nls” package and the best fit based on Akaike Information Criterion (AIC) was plotted in the graphs. T-tests were performed to identify differences between N content at different heights between monospecific-dominated and mixed stands.

To understand what drives the N allocation in the canopy we assembled multi-predictor linear models to test whether the N allocation coefficients (*K*_*N-F*_ and *K*_*N-G*_) were affected by the type of stand (monospecific-dominated vs. mixed stands), species richness (SR), FD_Q_-N, FRic, FEve, total N content per ground surface area (N_G_), and total N content per leaf area (N_F_), with the soil sand content as covariable (see Table 1 for a list of all abbreviations). To account for potential effects of N reallocation in vegetative and reproductive organs on the K_N_-values, we also included N_R_ (the N found in reproductive organs such as inflorescences) and stem biomass as covariables.

As predictors for NEE we tested the impact of the N allocation coefficients alongside N_G_ and N_F_, total leaf biomass (F_BM_) and the LAI, with soil sand content, cuvette measured radiation and temperature as covariables. Since in the Jena Experiment the soil texture was used to define blocks^46^, here we took into account any block-related effects by introducing the sand content (decreasing from 45% in block 1 to 5% in block 4) as a covariable in all models. All initial/maximal models were simplified to achieve most parsimonious models using the “glmulti” statistical package^52^ for automated model selection and model-averaging. This package offers the possibility to identify the most parsimonious models (including parameter interactions) based on Akaike Information Criterion adjusted for sample size (AICc)^53^.

## Electronic supplementary material

Suppmementary information
