# Supplementary material for: Top canopy nitrogen allocation linked to increased grassland carbon uptake in stands of varying species richness
Source: Sci Rep. 2017 Aug 16;7:8392. doi: 10.1038/s41598-017-08819-9 (PMC5559525; doi:10.1038/s41598-017-08819-9)

Supporting information for the article entitled "Top canopy nitrogen allocation linked to increased grassland carbon uptake in stands of varying species richness" by

Alexandru Milcu, Arthur Gessler, Christiane Roscher, Laura Rose, Zachary Kayler, Dörte Bachmann, Karin Pirhofer-Walzl, Saša Zavadlav, Lucia Galiano, Tina Buchmann, Michael Scherer-Lorenzen and Jacques Roy

This document contains:

1) **Supplementary Table S1**
2) **Supplementary Figures S1-S8**

Table S1. Table showing the species composition, estimated species cover, realized species richness (RSR) and species with a threshold surface cover higher than 15% (SR15).

**Species no./ Plot ID**	**1**	**2**	**3**	**4**	**5**	**6**	**7**	**8**	**9**	**10**	**11**	**12**	**13**	**14**	**15**	**16**	**17**	**18**	**19**	**20**	**21**
RSR	12	13	19	9	7	11	13	16	13	13	10	12	9	12	15	13	15	10	6	14	4
SR15	4	3	4	2	3	5	3	3	3	2	1	3	1	1	2	2	3	1	1	3	1
Achillea millefolium	0	10	0	0	0	0	0	0	0	0	0	0	0	80	0	0	0	0	0	0	0
Ajuga reptans	0	0	0	0	0	0	0	0	0	0	0	0	1	0	0	0	0	0	0	0	0
Alopecurus pratensis	0	0	0	0	0	0	0	0	0	0	0	0	1	0	1	1	1	1	0	0	1
Arrhenatherum elatius	2	2	10	10	0	40	0	20	20	30	90	1	1	10	50	20	30	10	0	2	0
Avenula pubescens	1	0	1	0	0	0	0	0	0	0	0	0	0	0	0	0	0	0	0	0	0
Bellis perennis	0	0	0	0	0	0	0	0	0	0	10	0	0	0	1	0	0	0	0	0	0
Bromus erectus	0	0	0	0	0	0	0	0	0	0	0	0	0	0	0	0	0	0	0	80	0
Bromus hordeaceus	0	0	0	0	0	1	1	0	0	0	0	0	0	0	0	0	0	0	0	0	0
Bromus sterilis	0	0	0	0	0	1	0	0	0	0	0	0	0	0	0	0	0	0	0	0	0
Centaurea jacea	0	0	2	0	0	0	0	0	0	0	0	0	0	0	0	0	0	0	0	0	0
Cerastium holosteoides	0	0	1	0	1	0	0	1	0	1	0	0	0	0	1	0	0	0	0	0	0
Cirsium arvense	0	2	0	0	0	2	0	0	0	0	0	0	0	0	0	0	0	0	2	2	0
Crepis biennis	0	30	2	20	1	0	2	0	0	0	2	0	0	0	10	2	0	10	0	0	0
Dactylis glomerata	0	0	0	0	90	0	80	0	1	0	0	1	0	1	0	10	0	0	0	1	0
Elytrigia repens	0	0	0	0	0	0	0	0	0	0	0	0	0	0	0	0	1	0	80	0	0
Festuca pratensis	0	0	0	0	0	0	0	0	0	0	0	20	0	0	1	0	0	0	0	0	0
Festuca rubra	0	0	0	0	0	0	0	0	0	0	0	0	0	0	0	0	2	0	0	0	0
Galium mollugo	20	0	0	10	20	20	0	2	0	40	0	2	0	1	60	1	0	2	0	0	90
Geranium pratense	30	0	30	80	0	50	0	10	20	10	0	0	10	10	0	0	0	10	0	1	0

Glechoma hederacea	0	2	0	0	20	0	0	2	2	0	0	2	0	0	0	0	0	0	0	0	0
Heracleum sphondylium	0	2	1	2	0	0	0	0	1	1	0	0	0	0	0	0	0	0	0	0	0
Holcus lanatus	10	0	0	0	0	0	0	0	0	10	0	0	0	0	0	2	0	0	0	0	0
Knautia arvensis	0	0	0	0	0	0	2	10	0	10	0	0	0	2	0	0	10	0	0	0	0
Lathyrus pratensis	10	0	10	0	10	0	0	2	1	0	0	40	0	1	1	0	2	80	10	0	0
Leontodon hispidus	0	0	0	0	0	0	1	0	0	0	2	0	0	0	0	0	0	0	0	0	0
Leucanthemum vulgare	0	0	1	0	0	0	0	0	0	0	0	0	0	1	1	0	1	0	0	1	0
Lolium perenne	0	0	0	0	0	0	0	0	0	0	0	1	0	0	0	0	0	0	0	0	0
Lotus corniculatus	10	0	0	0	0	0	0	0	0	0	0	0	0	2	0	60	30	0	0	0	0
Medicago x varia	0	0	30	0	0	20	0	30	40	0	0	0	0	0	0	0	0	0	0	0	0
Onobrychis viciifolia	0	60	0	0	0	0	0	0	0	0	0	0	0	0	0	0	0	0	0	0	0
Pastinaca sativa	0	0	0	0	0	0	0	0	0	0	0	0	0	0	0	0	2	0	0	10	0
Pimpinella major	0	30	20	0	0	0	0	20	10	0	0	0	0	10	0	0	0	2	0	0	0
Plantago lanceolata	0	0	2	0	0	1	2	2	0	2	2	10	1	0	2	10	2	0	0	1	0
Plantago media	0	0	0	0	0	0	0	0	0	0	0	30	2	0	0	2	0	0	0	0	0
Poa pratensis	1	0	0	0	0	0	0	0	0	0	0	0	0	0	0	0	0	0	0	0	0
Poa trivialis	20	10	10	10	2	30	20	10	2	10	2	10	2	2	10	2	10	10	10	20	2
Primula veris	0	0	0	0	0	0	0	2	0	0	0	0	0	0	0	0	0	0	0	0	0
Prunella vulgaris	0	0	0	0	0	0	0	0	0	0	2	0	0	0	0	0	2	0	0	0	0
Ranunculus acris	10	0	2	2	0	0	0	0	1	10	0	0	0	0	0	1	0	0	0	0	0
Ranunculus repens	0	0	0	0	0	0	0	0	0	0	0	0	0	0	0	0	0	0	0	2	0
Rumex acetosa	0	2	0	0	0	0	0	2	10	0	0	0	0	0	0	0	0	1	1	20	0
Silene vulgaris	20	0	0	0	0	0	0	0	0	0	0	0	0	0	0	0	0	0	0	0	0
Taraxacum officinale	2	2	0	0	0	2	1	10	1	2	0	0	2	0	0	10	2	0	0	10	2
Trifolium dubium	0	0	2	0	0	0	2	0	0	0	0	0	0	0	0	0	0	0	0	0	0
Trifolium pratense	0	1	0	0	0	0	0	0	0	1	0	0	0	0	10	0	20	0	0	0	0
Trifolium repens	0	0	0	1	0	0	0	0	0	0	0	0	0	0	0	0	0	0	0	0	0
Trisetum flavescens	0	0	20	0	0	0	10	10	10	0	0	0	0	0	1	0	0	0	0	0	0
Veronica arvensis	0	1	1	0	0	0	1	0	0	0	1	0	0	0	0	0	0	0	0	0	0
Veronica chamaedrys	0	0	0	0	0	10	0	0	0	0	0	0	0	0	0	0	0	0	0	0	0
Vicia angustifolia	0	0	2	0	0	0	1	0	0	0	1	1	0	0	2	1	1	0	0	0	0
Vicia cracca	0	0	2	0	0	0	20	1	0	0	10	2	90	2	1	0	0	1	2	2	0
Vicia sepium	0	0	0	1	0	0	0	0	0	10	0	0	0	0	0	0	0	0	0	2	0

Supplementary Fig. S1. Light attenuation profiles and coefficients (K_L) for all canopies.

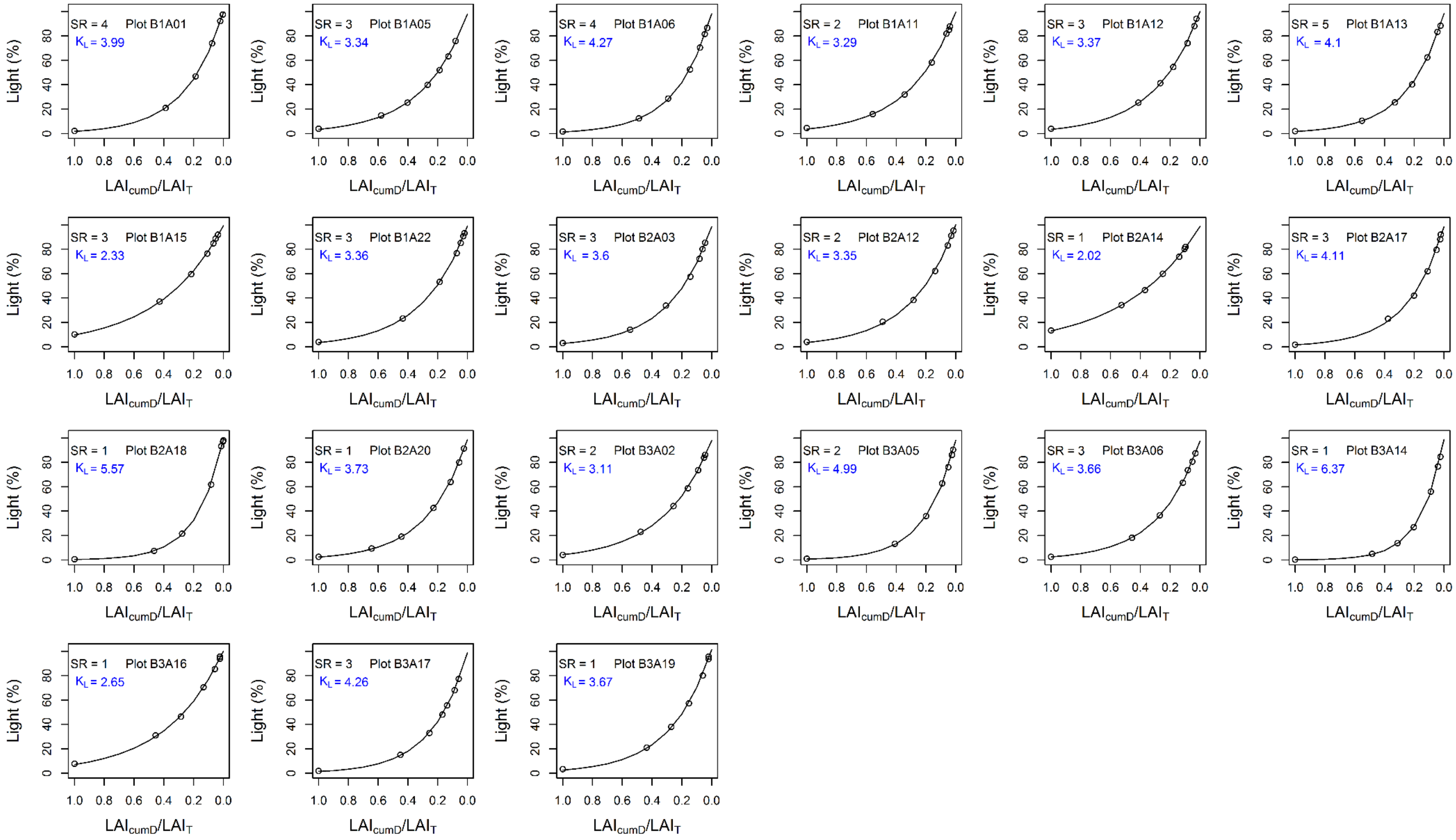

Supplementary Fig. S2. Nitrogen allocation coefficients based on the N content per foliar surface area ($K_{N\text{-}F}$) for all canopies.

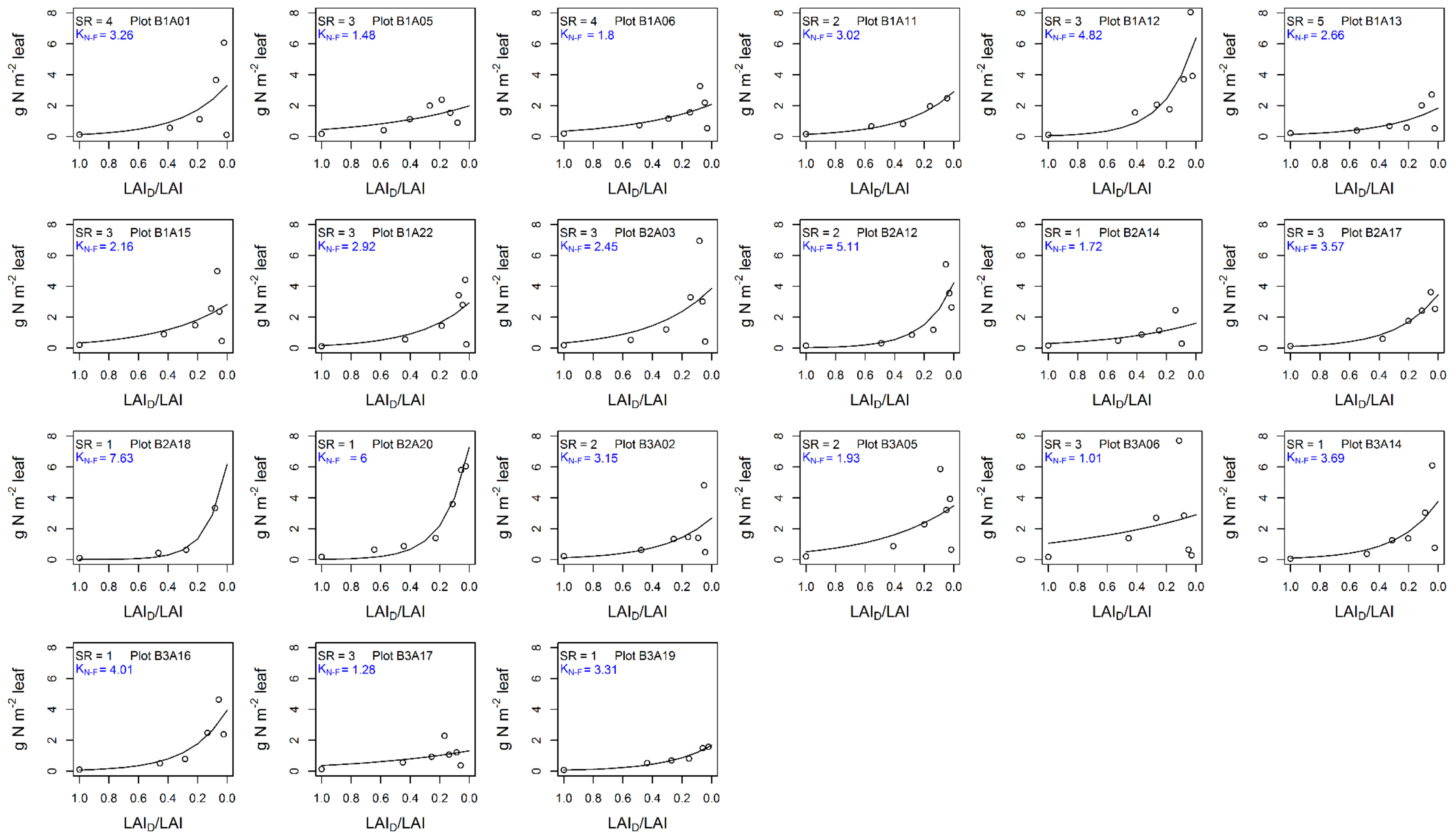

Supplementary Fig. S3. Nitrogen allocation coefficients based on the N content per surface ground area ($K_{N\text{-}G}$) for all canopies.

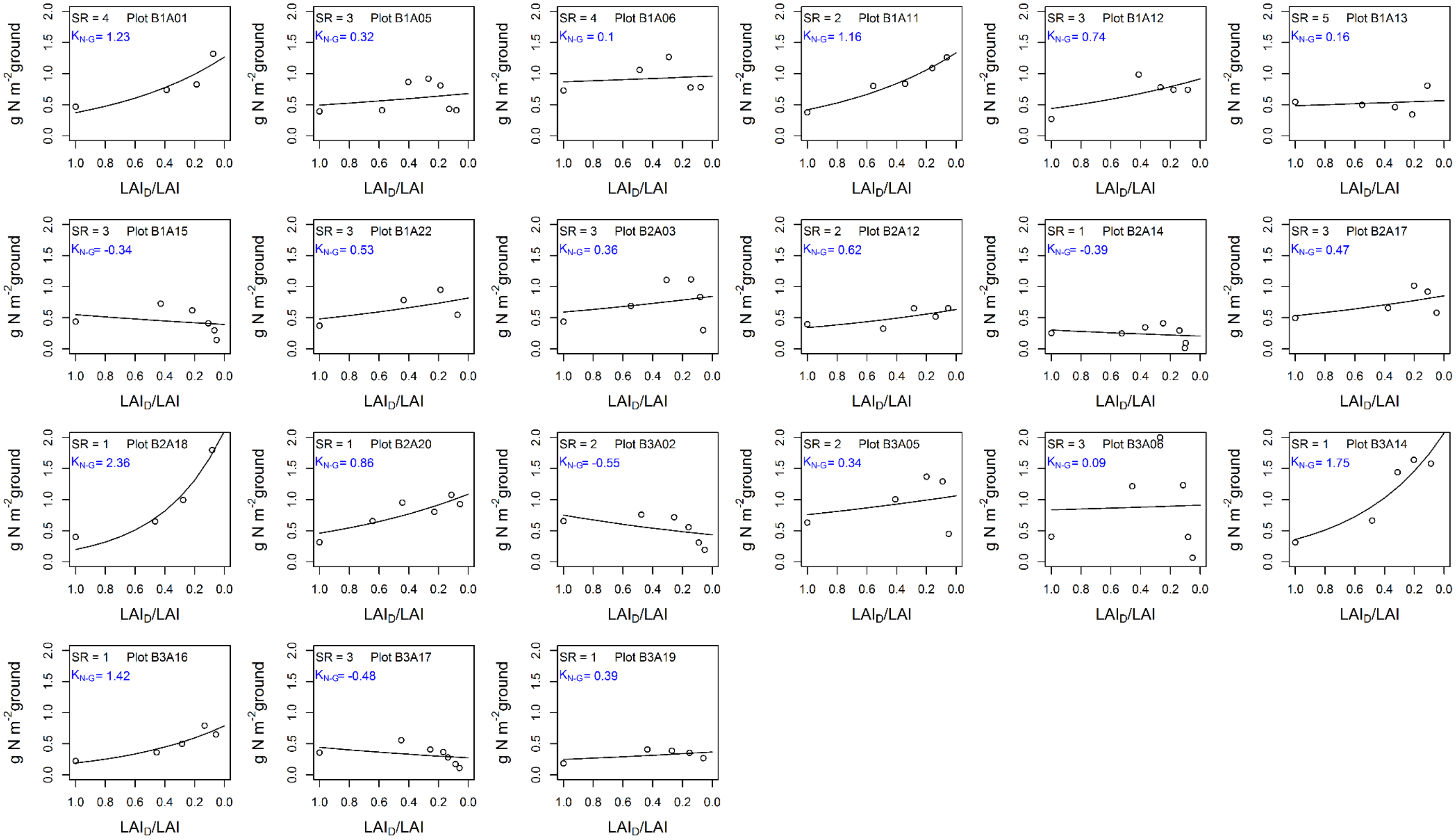

Supplementary Fig. S4. Correlation matrix of main predictors and response variables (see methods for abbreviations).

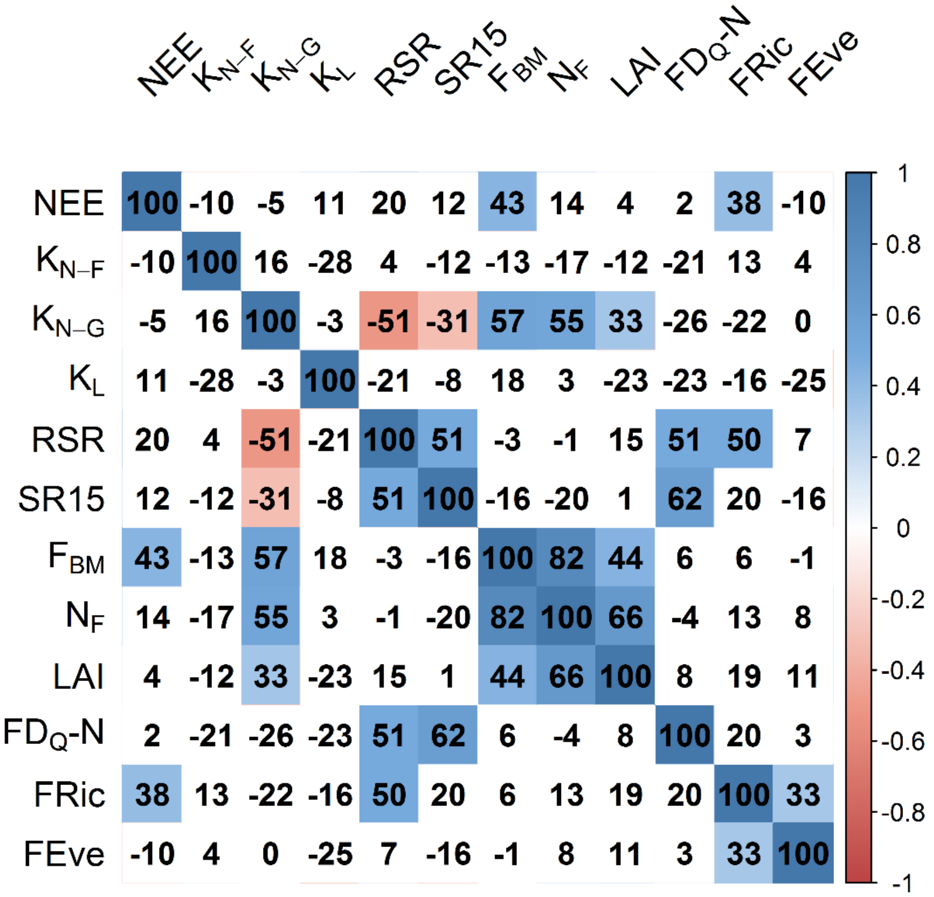

Figure S5 Scatterplots and regression lines depicting the relationship between realized species riches (RSR) and species richness calculated with a threshold cover > 55% (SR15) and CO_2 net ecosystem exchange (NEE), nitrogen distribution coefficients calculated per surface foliar area (K_{N-F}) and ground surface area (K_{N-G}).

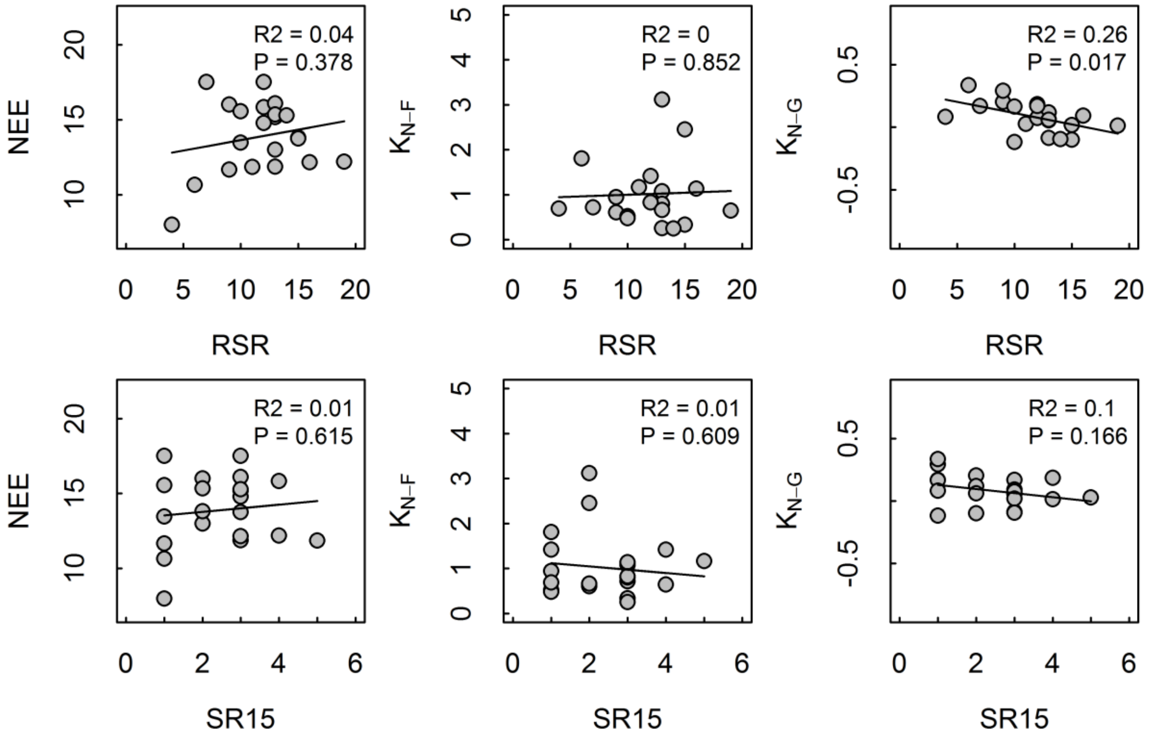

Figure S6. Scatterplots and regression lines depicting the relationship between several diversity metrics (Simpson , Shannon and evenness) and CO_2 net ecosystem exchange (NEE) and nitrogen distribution coefficients calculated per surface foliar area (K_{N-F}) and ground surface area (K_{N-G}).

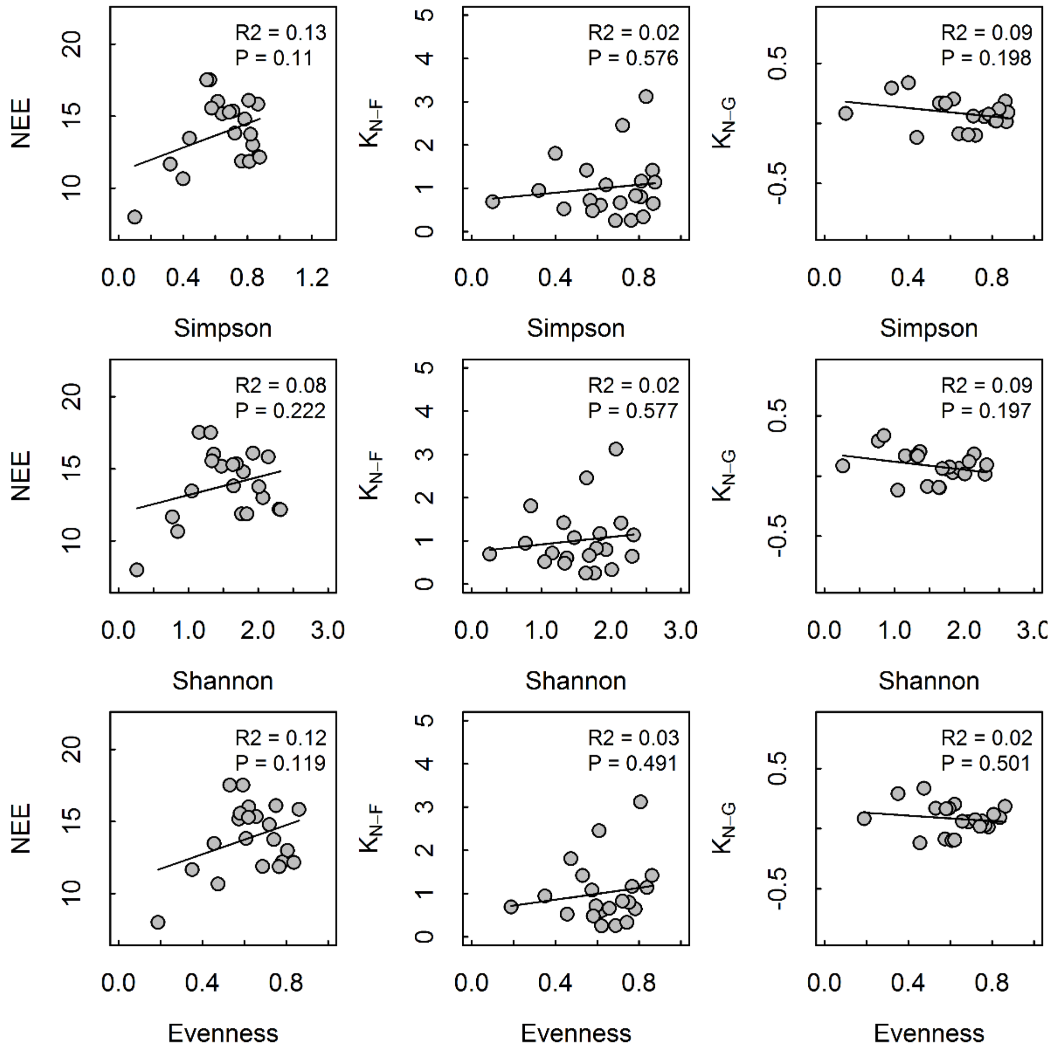

Figure S7. Scatterplot and correlation matrix depicting the relationships between nitrogen use efficiency (NUE), light use efficiency (LUE) and their most important predictors (See Table 1 for a complete list of abbreviations). The line in the scatterplots represents a locally weighted scatter-plot smoother (loess) fitting.

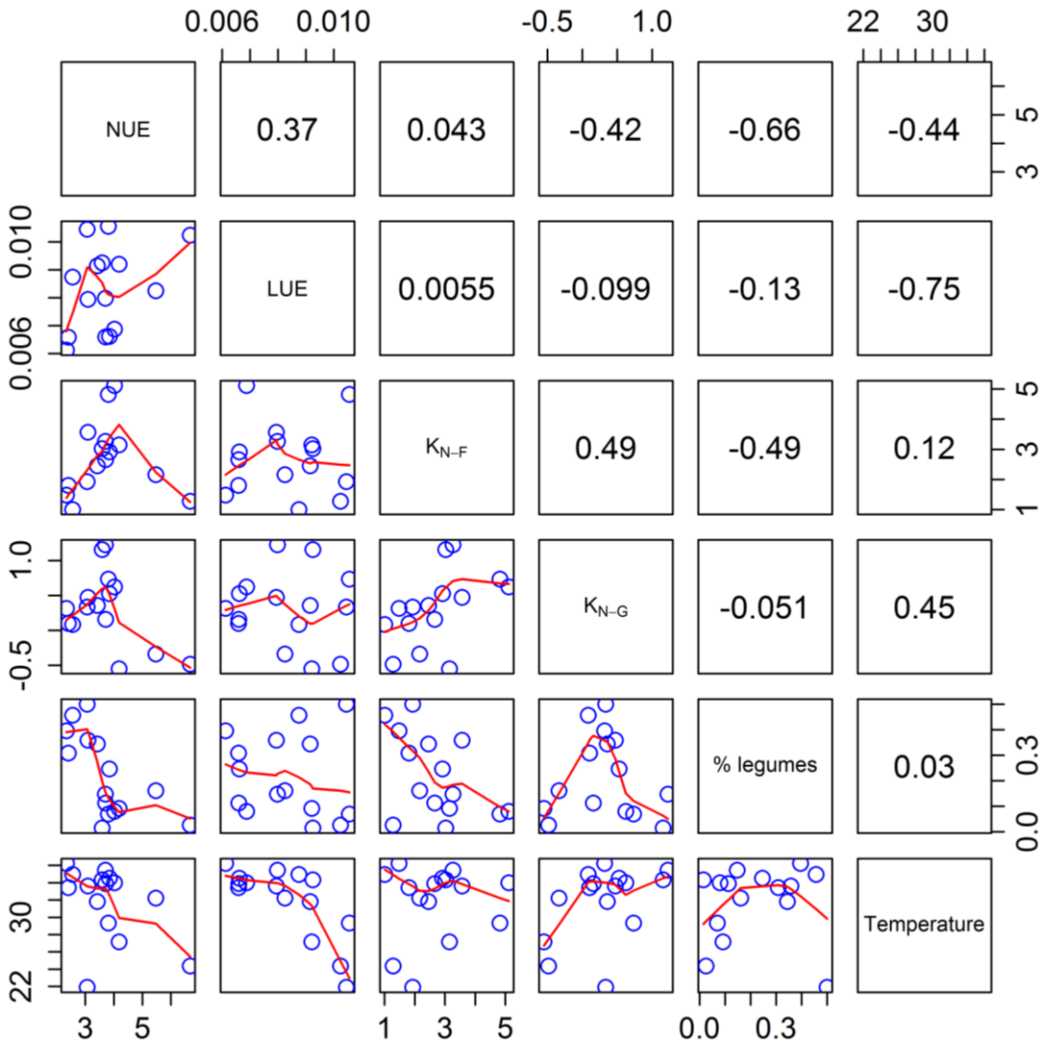

Figure S8. Relationships between leaf nitrogen concentration and leaf photosynthetic capacity (Amax) in eight species present in our study (Arr ela = *Arrhenatherum elatius*; Dac glo = *Dactylis glomerata*; Ely rep = *Elytrigia repens*; Ger pra = *Geranium pratense*; Kna arv = *Knautia arvensis*; Leu vul = *Leucanthemum vulgare*; Ono vic = *Onobrychis viciifolia*; Tri pra = *Trifolium pratense*; Vic sep = *Vicia sepium*).

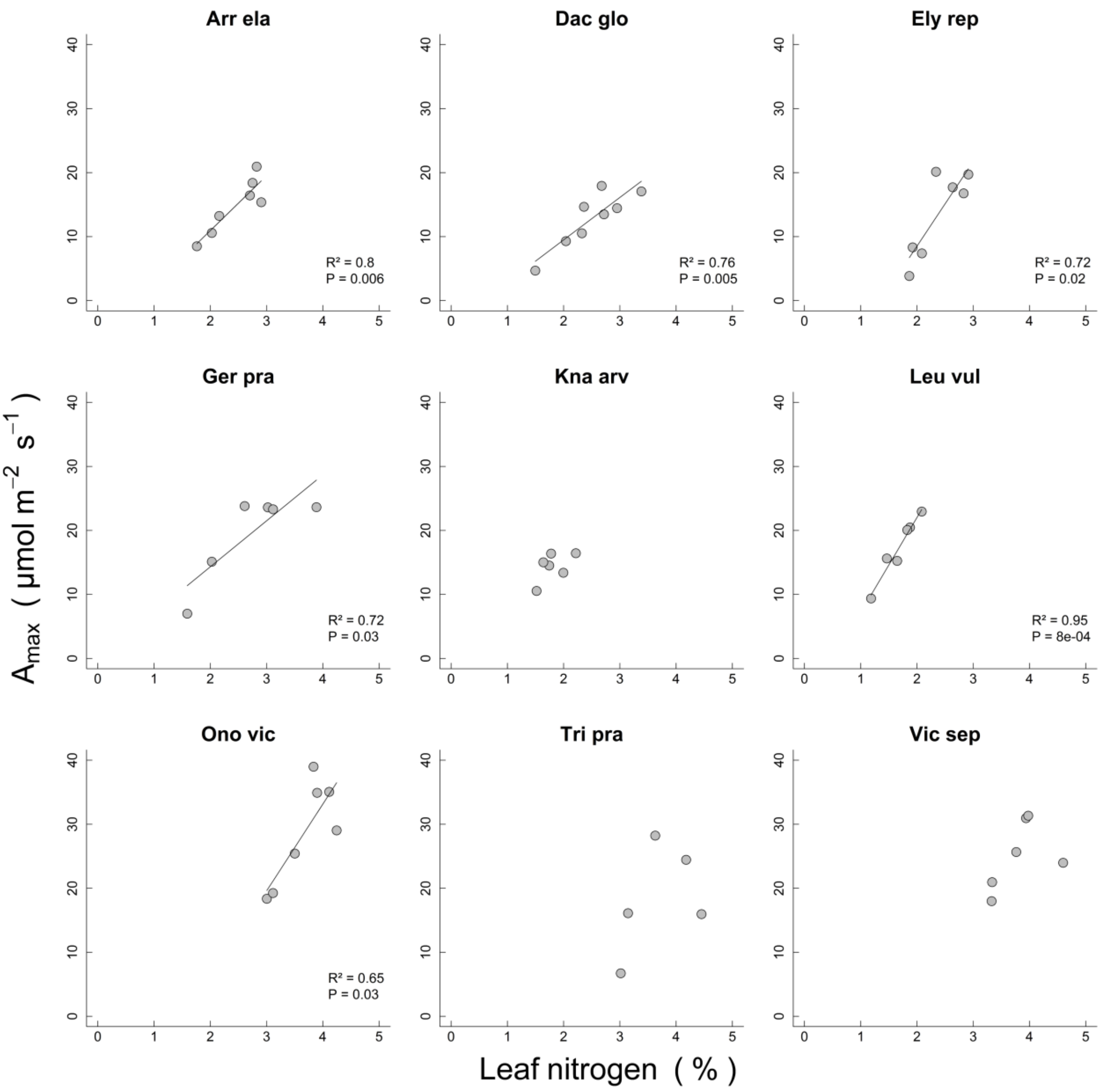

Supplement: Supplementary file 1 — Suppmementary information [file 41598_2017_8819_MOESM1_ESM.pdf]
